# Supplementary material for: Coupling of cell shape, matrix and tissue dynamics ensures embryonic patterning robustness
Source: Nat Cell Biol. 2025 Feb 18;27(3):408–23. doi: 10.1038/s41556-025-01618-9 (PMC11906357; doi:10.1038/s41556-025-01618-9)
Supplement: Supplementary file 1 — Reporting Summary [file 41556_2025_1618_MOESM1_ESM.pdf]

nature portfolio

Corresponding author(s): Takashi Hiiragi

Last updated by author(s): Nov 19, 2024

Reporting Summary

Nature Portfolio wishes to improve the reproducibility of the work that we publish. This form provides structure for consistency and transparency in reporting. For further information on Nature Portfolio policies, see our Editorial Policies and the Editorial Policy Checklist.

Statistics

For all statistical analyses, confirm that the following items are present in the figure legend, table legend, main text, or Methods section.

n/a	Confirmed	
☐	☒	The exact sample size (*n*) for each experimental group/condition, given as a discrete number and unit of measurement
☐	☒	A statement on whether measurements were taken from distinct samples or whether the same sample was measured repeatedly
☐	☒	The statistical test(s) used AND whether they are one- or two-sided<br>*Only common tests should be described solely by name; describe more complex techniques in the Methods section.*
☐	☒	A description of all covariates tested
☐	☒	A description of any assumptions or corrections, such as tests of normality and adjustment for multiple comparisons
☐	☒	A full description of the statistical parameters including central tendency (e.g. means) or other basic estimates (e.g. regression coefficient) AND variation (e.g. standard deviation) or associated estimates of uncertainty (e.g. confidence intervals)
☐	☒	For null hypothesis testing, the test statistic (e.g. *F*, *t*, *r*) with confidence intervals, effect sizes, degrees of freedom and *P* value noted<br>*Give P values as exact values whenever suitable.*
☒	☐	For Bayesian analysis, information on the choice of priors and Markov chain Monte Carlo settings
☒	☐	For hierarchical and complex designs, identification of the appropriate level for tests and full reporting of outcomes
☒	☐	Estimates of effect sizes (e.g. Cohen's *d*, Pearson's *r*), indicating how they were calculated

Our web collection on statistics for biologists contains articles on many of the points above.

Software and code

Policy information about availability of computer code

Data collection	Zen (Zeiss, 2012), Dikeria (Biro and Maitre, 2015)
Data analysis	FIJI v2.14.0 (RRID: SCR_002285), Imaris v9.7.2 (RRID: SCR_007370), Napari v0.4.17, Mov-IT (Faure et al., 2016), Nuclear Detection and Tracking (Fabreges et al., 2024), Python v3.9 (RRID:SCR_008394), SciPy v1.7.1 (RRID:SCR_008058), Scikit-learn (RRID:SCR_019053), Wolfram Mathematica (RRID:SCR_014448)

For manuscripts utilizing custom algorithms or software that are central to the research but not yet described in published literature, software must be made available to editors and reviewers. We strongly encourage code deposition in a community repository (e.g. GitHub). See the Nature Portfolio guidelines for submitting code & software for further information.

Data

Policy information about availability of data

All manuscripts must include a data availability statement. This statement should provide the following information, where applicable:

- Accession codes, unique identifiers, or web links for publicly available datasets
- A description of any restrictions on data availability
- For clinical datasets or third party data, please ensure that the statement adheres to our policy

Source data are provided with this manuscript. All other data supporting the findings of this study are available within the manuscript. Image data can be obtained from the corresponding author upon request. Previously published images of human embryonic material that were re-analysed here were obtained from

doi.org/10.1242/dev.201522.

Research involving human participants, their data, or biological material

Policy information about studies with human participants or human data. See also policy information about sex, gender (identity/presentation), and sexual orientation and race, ethnicity and racism.

Reporting on sex and gender	No human research participants were used.
Reporting on race, ethnicity, or other socially relevant groupings	Not Applicable
Population characteristics	Not Applicable
Recruitment	Not Applicable
Ethics oversight	Not Applicable

Note that full information on the approval of the study protocol must also be provided in the manuscript.

Field-specific reporting

Please select the one below that is the best fit for your research. If you are not sure, read the appropriate sections before making your selection.

☒ Life sciences ☐ Behavioural & social sciences ☐ Ecological, evolutionary & environmental sciences

For a reference copy of the document with all sections, see nature.com/documents/nr-reporting-summary-flat.pdf

Life sciences study design

All studies must disclose on these points even when the disclosure is negative.

Sample size	Sample size was based on prior literature using similar experimental paradigms:<br><br>Ryan, A. Q., Chan, C. J., Graner, F., & Hiiragi, T. (2019). Lumen expansion facilitates epiblast-primitive endoderm fate specification during mouse blastocyst formation. Developmental cell, 51(6), 684-697.<br><br>Chan, C. J., Costanzo, M., Ruiz-Herrero, T., Mönke, G., Petrie, R. J., Bergert, M., ... & Hiiragi, T. (2019). Hydraulic control of mammalian embryo size and cell fate. Nature, 571(7763), 112-116.
Data exclusions	1. In the cell tracking analysis in isolated ICMs, all cells in the ICM were inspected, cells that could not be tracked with confidence (<1% cells) were excluded from the lineage trees.<br>2. In the embryo aggregation experiments, embryos that failed to aggregate and those that did not form a singular blastocyst cavity were excluded from analysis.<br>3. For quantification of ectopic ICM cells in size-manipulated mouse blastocysts, small-sized blastocysts lacking an ICM were excluded from the analysis.<br>4. In size-manipulated mouse blastocysts, small-sized blastocysts lacking an ICM and those lacking an ICM-cavity fluid interface were excluded from the analysis to measure ICM geometrical dimensions.<br>5. In the implanted bead experiments, embryos where the bead was not successfully implanted in the ICM were excluded.
Replication	All data were generated in triplicates, and replication attempts were successful.
Randomization	Mice of the desired genotype were pooled together and randomly allocated for experiments. This is not relevant for monkey blastocysts as we do not have multiple experimental groups, all monkey embryos obtained from intracytoplasmic sperm injection of oocytes were fixed at late blastocyst stage.
Blinding	For experiments with knock-out mutant embryos, the researchers were blinded to the genotype of the samples during immunofluorescence imaging and data analysis as single-embryo genotyping by PCR was performed retrospectively.

Reporting for specific materials, systems and methods

We require information from authors about some types of materials, experimental systems and methods used in many studies. Here, indicate whether each material, system or method listed is relevant to your study. If you are not sure if a list item applies to your research, read the appropriate section before selecting a response.

Materials & experimental systems

n/a	Involved in the study
☐	☒ Antibodies
☒	☐ Eukaryotic cell lines
☒	☐ Palaeontology and archaeology
☐	☒ Animals and other organisms
☒	☐ Clinical data
☒	☐ Dual use research of concern
☒	☐ Plants

Methods

n/a	Involved in the study
☒	☐ ChIP-seq
☒	☐ Flow cytometry
☒	☐ MRI-based neuroimaging

Antibodies

Antibodies used

Primary antibodies used in this study were:
GATA6 (R&D systems, AF1700),
GATA4 for mouse embryos (R&D systems, BAF2606),
GATA4 for monkey embryos (Cell Signalling, 36966S),
SOX2 (Cell Signaling, 23064),
bi-phosphorylated myosin regulatory light chain (ppMRLC) (Cell Signaling, 3674),
Laminin (Novus Biologicals, NB300-14422),
NANOG (ReproCell, RCAB002P-F),
PKC-lambda (Santa Cruz Biotechnology, sc-17837),
PKC-zeta (Santa Cruz Biotechnology, sc-17781),
Integrinb1 clone MB1.2 (Millipore, MAB1997),
active Integrinb1 (9EG7, BD Bioscience, 553715),
E-cadherin (Sigma, U3254),
RFP/tdTomato (Rockland, 600-401-379)
RFP/tdTomato (Chromotek, 5f8)
Alexa Fluor 647-conjugated Oct3/4 (Santa Cruz, sc-5279 AF647).

Secondary antibodies used in this study were:
donkey anti-goat IgG Alexa Fluor 488 (Invitrogen, A11055),
donkey anti-rabbit IgG Alexa Fluor Plus 488 (Thermo Fisher Scientific, A32790),
donkey anti-rabbit IgG Alexa Fluor 546 (Invitrogen, A10040),
donkey anti-mouse IgG Alexa Fluor 555 (Invitrogen, A31570),
donkey anti-rabbit IgG Alexa Fluor 647 (Invitrogen, A31573),
donkey anti-mouse Cy5 (Jackson ImmunoResearch, 715-175-150),
donkey anti-rat Cy5 (Jackson ImmunoResearch, 712-175-153),
donkey anti-rabbit IgG Alexa Fluor Plus 488 (Thermo Fisher Scientific, A32790).

Validation

GATA6 (R&D systems, AF1700), was validated by the manufacturer using WB and ELISA.
GATA4 (R&D systems, BAF2606), was validated by the manufacturer using WB.
GATA4 (Cell Signalling, 36966S) was validated by the manufactures using WB and IF.
Alexa Fluor 647-conjugated Oct3/4 (Santa Cruz, sc-5279 AF647) was validated by the manufacturer using WB.
SOX2 (Cell Signaling, 23064), was validated by the manufacturer using WB, IP, IF and ChIP.
bi-phosphorylated myosin regulatory light chain (ppMRLC) (Cell Signaling, 3674), was validated by the manufacturer using WB.
Laminin (Novus Biologicals, NB300-14422), was validated by the manufacturer using WB, IF.
NANOG (ReproCell, RCAB002P-F), was validated by the manufacturer using IF.
PKC-lambda (Santa Cruz Biotechnology, sc-17837), was validated by the manufacturer using WB.
PKC-zeta (Santa Cruz Biotechnology, sc-17781), was validated by the manufacturer using WB, IF.
Integrinb1 (Millipore, MAB1997), was validated by the manufacturer using WB.
active Integrinb1 (9EG7, BD Bioscience, 553715) was validated by the manufacturer using IHC.
E-cadherin (Sigma, U3254) was validated by the manufacturer using IHC and IP.
RFP/tdTomato (Rockland, 600-401-379) was validated by the manufacturer using IHC, IF and WB
RFP/tdTomato (Chromotek, 5f8) was validated by the manufacturer using IF and ELISA

Animals and other research organisms

Policy information about studies involving animals; ARRIVE guidelines recommended for reporting animal research, and Sex and Gender in Research

Laboratory animals

Mouse, species: Mus musculus, strain: C57BL/6xC3H F1 hybrids, age: between 8-30 weeks of age. Details of genetically-modified mice (GM) are provided in the Methods section of the manuscript.
Monkey, species: Macaca fascicularis (monkeys do not have strain information as in inbred mouse strains), ages ranging between 6 to 11 years.

Wild animals

The study did not involve wild animals.

Reporting on sex

Sex does not influence the findings reported in the study.

Field-collected samples	The study did not involve field-collected samples.
Ethics oversight	All mouse-related animal work performed at the Laboratory Animal Resources (LAR) Facility at European Molecular Biology Laboratory (EMBL) was done with permission from the Institutional Animal Care and Use Committee (IACUC) overseeing the operation (IACUC number TH11 00 11). LAR facilities operate according to Federation for Laboratory Animal Science Associations (FELASA) guidelines and recommendations.<br><br>At the Hubrecht Institute animal facility, mice were housed according to institutional guidelines, and procedures were performed in compliance with Standards for Care and Use of Laboratory Animals with approval from the Hubrecht Institute ethical review board. Animal experiments were approved by the Animal Experimentation Committee (DEC) of the Royal Netherlands Academy of Arts and Sciences.<br><br>Monkey animal work was appropriately performed by following the Animal Research:Reporting in Vivo Experiments (ARRIVE) guidelines developed by the National Centre for the Replacement, Refinement & Reduction of Animals in Research (NC3Rs), and also by following “The Act on Welfare and Management of Animals” from Ministry of the Environment, “Fundamental Guidelines for Proper Conduct of Animal Experiment and Related Activities in Academic Research Institutions” under the jurisdiction of the Ministry of Education, Culture, Sports, Science and Technology, and “Guidelines for Proper Conduct of Animal Experiments” from Science Council of Japan. All animal experimental procedures were approved by the Animal Care and Use Committee of Shiga University of Medical Science (approval number: 2021-10-4)

Note that full information on the approval of the study protocol must also be provided in the manuscript.
